# Low levels of sibship encourage use of larvae in western Atlantic bluefin tuna abundance estimation by close-kin mark-recapture

**DOI:** 10.1038/s41598-022-20862-9

**Published:** 2022-11-03

**Authors:** Jan R. McDowell, Mark Bravington, Peter M. Grewe, Matthew Lauretta, John F. Walter, Shane M. Baylis, Thierry Gosselin, Estrella Malca, Trika Gerard, Akihiro Shiroza, John T. Lamkin, Ellen E. Biesack, Glenn Zapfe, Walter Ingram, Campbell Davies, Clay Porch

**Affiliations:** 1 Virginia Institute of Marine Science, William and Mary, Gloucester Point, VA USA; 2 CSIRO Oceans and Atmosphere, Hobart, TAS Australia; 3 National Oceanic Atmospheric Administration, National Marine Fisheries Service, Miami, FL USA; 4 Unaffiliated, Toronto, Canada; 5 Cooperative Institute for Marine and Atmospheric Studies, University of Miami, Miami, FL USA

**Keywords:** Conservation biology, Ecosystem ecology, Population dynamics, Genotype, Population genetics, Sequencing, Marine biology

## Abstract

Globally, tunas are among the most valuable fish stocks, but are also inherently difficult to monitor and assess. Samples of larvae of Western Atlantic bluefin tuna *Thunnus thynnus* (Linnaeus, 1758) from standardized annual surveys in the northern Gulf of Mexico provide a potential source of “offspring” for close-kin mark-recapture (CKMR) estimates of abundance. However, the spatial patchiness and highly skewed numbers of larvae per tow suggest sampled larvae may come from a small number of parents, compromising the precision of CKMR. We used high throughput genomic profiling to study sibship within and among larval tows from the 2016 standardized Gulf-wide survey compared to targeted sampling carried out in 2017. Full- and half-siblings were found within both years, with 12% of 156 samples in 2016 and 56% of 317 samples in 2017 having at least one sibling. There were also two pairs of cross cohort half-siblings. Targeted sampling increased the number of larvae collected per sampling event but resulted in a higher proportion of siblings. The combined effective sample size across both years was about 75% of the nominal size, indicating that Gulf of Mexico larval collections could be a suitable source of juveniles for CKMR in Western Atlantic bluefin tuna.

## Introduction

Commercial tuna fisheries produce an estimated $42 billion in economic value and are a major source of global protein food supply^1^. The International Commission for the Conservation of Atlantic Tunas manages Atlantic bluefin tuna, *Thunnus thynnus* (Linnaeus, 1758; BFT), populations by geographic area, with a jurisdictional boundary divided by 45° west longitude. This management strategy is based on the fact that BFT are known to exhibit some level of fidelity to major spawning grounds in the Gulf of Mexico (GoM) and Mediterranean Sea (Med) as evidenced by conventional and satellite tagging (Hanke et al.^2^ and references therein), organochlorine tracers^3^, otolith chemistry^4,5^ and genetics^6^. However, a substantial body of work has demonstrated that BFT tuna originating from GoM and from Med mix extensively on foraging grounds^7–11^, where much of the catch is taken. This highly migratory behavior, pan-oceanic distribution, and stock mixing, combined with the high cost of surveying fish populations in the open ocean, has made accurate assessment of Atlantic bluefin tuna stocks challenging.

Recent technological and analytical developments have provided a compelling rationale to explore the feasibility of close-kin mark-recapture (CKMR) for western BFT. First, DNA profiling using high-throughput genotyping-by-sequencing of reduced representation libraries^12,13^ can identify individual genotypes, which can be used to accurately infer full-sibling, half-sibling, and parent–offspring relationships in the absence of a known pedigree. CKMR harnesses the tag-recapture nature of the Parent–Offspring Pair (POP) relationship, i.e., that one offspring ‘tags’ its two parents, to allow estimation of absolute adult abundance, as well as other demographic parameters such as fecundity and mortality rate^14–16^. The two tags from each juvenile sample can be recaptured either directly, if the parent is found amongst a sample of adults, or indirectly if a sibling is found amongst a sample of juveniles from a different cohort^14–16^. Unlike with conventional mark-recapture, all sampling events can be lethal. Recent CKMR applications have provided estimates of absolute abundance for Southern bluefin tuna^17^, white sharks^18^, brook trout^19^, and thornback rays^20^. The success of these studies motivates application to other cases, especially where standard population assessment methods (e.g., conventional tagging for mark-recapture) have been challenged by data limitations and the difficulty of surveying highly migratory species. This is the case for BFT given the failure of recent assessments to estimate biomass-based reference points^21^, and associated uncertainty attributed to stock mixing of western (GoM) and eastern (Med) origin fish in the North Atlantic feeding aggregations.

Given an adult abundance on the order of approximately 10^5^ (a ballpark figure from recent attempts at stock assessment), CKMR for western BFT will require several thousand samples spread over a few years and including both adults and juveniles to yield enough POPs for a usefully precise result^14^. As for the adults (i.e., potential parents), life history studies have demonstrated spawning seasonality, site fidelity, and specific migration routes of GoM-spawning BFT^22–25^. Adults enter the GoM between November and May, with a spawning peak in the northern Gulf during April through May^24,25^. After spawning, fish migrate to foraging hotspots in the Northwest Atlantic Ocean^26,27^, where they form mixed stock feeding aggregations with eastern BFT and are targeted by multiple international fishing fleets. The National Oceanic and Atmospheric Administration (NOAA) conducts biological monitoring of BFT caught in U.S. fisheries during June to October, and Fisheries and Oceans Canada samples large BFT caught on New Brunswick and Nova Scotia foraging grounds. Although these samples are from mixed-stock feeding aggregations^28^, they can now be genetically assigned into eastern (Med) and western (GoM) Atlantic origin; 81% of known GoM origin BFT samples and 85% of known Med origin BFT samples were correctly assigned based on genetic markers^28,29^, enabling the use of GoM-assigned samples as the adults in CKMR^30^.

The second major obstacle for western BFT CKMR is where to get the juvenile samples (i.e., potential offspring). It is not essential for the juveniles in CKMR to be very young, but CKMR abundance estimates are intrinsically back-dated to the birth-years of the juvenile samples (i.e., when their parents were “tagged”). Hence, for an up-to-date estimate it is desirable to use fairly young juveniles, and it also is important to have reasonably accurate age estimates, although some uncertainty can be tolerated (e.g.,^18^). With western BFT, it has not been possible to organize reliable large-scale sampling of fish in the 1–4 yo age range during the past 20 + years. An alternative juvenile sample could be obtained from larvae that are currently collected as part of NOAA’s annual surveys, which are used to estimate relative-spawning-biomass-based reference points for GoM BFT^21^. These larvae represent an invaluable source of DNA from individuals caught during their first two weeks of life and thus represent a pure sample of the western BFT spawning stock. By pairing these annual larval collections with adult fish caught in Atlantic mixed stock fisheries and genetically identified as western Atlantic stock fish, we can potentially obtain the parent–offspring and cross-cohort sibling comparisons required to estimate absolute abundance of the adult western BFT spawning stock through CKMR^30^.

Two issues need to be addressed when using the larvae instead of older juveniles as the potential offspring in CKMR. The first is whether larval samples will yield a sufficient quantity and quality of DNA to allow reliable genotyping of enough loci to permit confident identification of siblings (i.e., full-sibling pairs (FSPs) and half-sibling pairs (HSPs), rather than just POPs). Although resolving HSP relationships requires more loci than POPs, recovery of cross-cohort siblings is extremely valuable for estimating average adult survival rates^31^. The second issue is more fundamental: larvae may be aggregated into sibling-groups from the same mating event, which could lower the number of statistically independent comparisons to the point where CKMR estimates become useless. Precise estimates of abundance from CKMR depend on having a good number of POPs from statistically independent comparisons, and this is constrained by how many parents are marked by the larval sample. Comparisons between one adult and several juveniles that are siblings are not independent because, after the first juvenile is compared to the adult, the outcomes of the other comparisons with its siblings are predictable. If 1000 larvae collectively mark only 6 distinct parents, then the number of statistically independent comparisons between larvae and adults is vastly reduced compared to what one might expect from an independently drawn juvenile sample of the same size.

There are a priori reasons to be concerned about possible high levels of sibship among western BFT larval samples. Active BFT spawning in the GOM occurs daily from mid-April to June^32,33^, a relatively short season compared to other tunas, with groups of individuals observed spawning together at the sea surface^34,35^. However, there has been no assessment of the proportion of individuals in these groups successfully participating in spawning events, and the number per event might be small. Furthermore, approximately 90% of the western BFT larvae collected during the annual surveys in the GoM come from only 10% of the larval tows, so a tow may not be sampling many spawning events. The larvae sampled in the current study were four to 11 days old and since larvae are most often found within the boundaries of anticyclonic features^36–38^, they have not had much opportunity to disperse since fertilization. Taken together this suggests the potential for a higher incidence of FSPs and HSPs within a limited set of tows relative to the cohort as a whole, as has been noted in other species^39^.

The current study evaluated whether BFT larvae collected during scientific surveys in the GoM can comprise an adequate juvenile sample for CKMR. We analyzed tail clips from hundreds of individual BFT larvae to assess whether the quality and quantity of DNA recovered was sufficient for use in high-throughput genotyping-by-sequencing applications. Next, we evaluated whether larvae collected in the GoM have levels of sibship that preclude genetic tagging of a sufficient number of adults for precise estimates of abundance based on CKMR. We demonstrated mathematically that bias is not a problem provided an appropriate CKMR model is formulated in the first place (in this case, taking account of adult size) and we developed formulae for effective sample size when sibship is high. We then compared the incidence of sibship from the spatially-spread-out 2016 larval survey to that in 2017, when a targeted BFT survey was used to maximize the number of larvae sampled, to test the relative efficacy of each sampling strategy for CKMR.

## Results

### BFT larval collections

**Figure 1:**
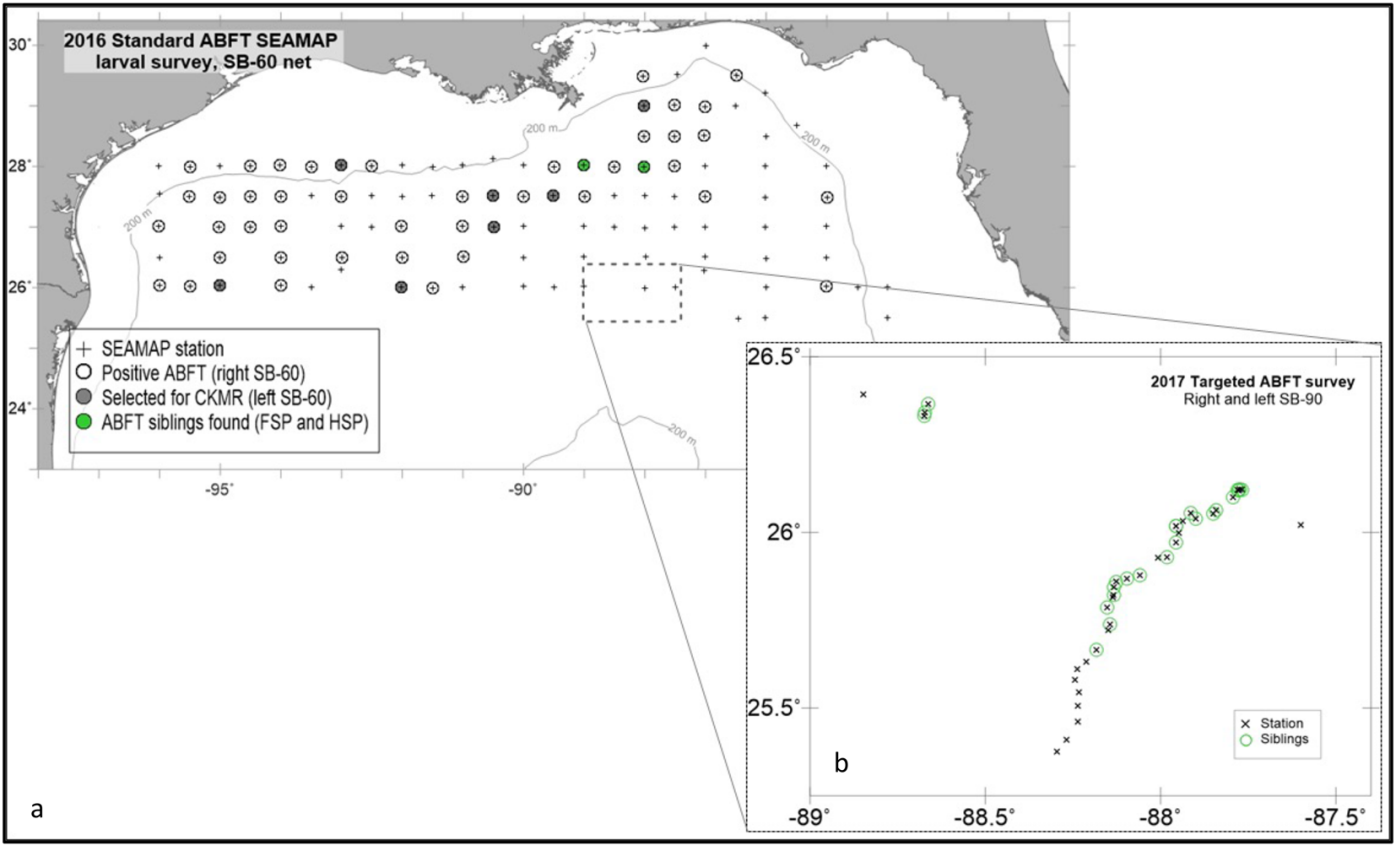
Larval Atlantic bluefin tuna (BFT) collected during two oceanographic surveys in the northern GoM, (**a**) 2016 standard ichthyoplankton survey (**b**) Insert: 2017 targeted larval survey. Stations are indicated with a + or x, in 2016, positive BFT stations that were selected for close kin mark recapture (CKMR) are displayed with closed symbols (gray filled circle). Surveys found to have siblings (full sibling pairs and half sibling pairs) are denoted by green symbols. The image was created in Surfer 9 (Golden Software LLC, Golden CO), https://www.goldensoftware.com/products/surfer.

In 2016, 600 BFT larvae were obtained from the left-side SB-60 net from the nine most larvae-rich right-side SB-60 stations, of which 345 were selected for genetic analysis (Table 1, Fig. 1a). In 2017, 872 BFT larvae were collected in 29 of 120 stations (22%) during the targeted survey (Table 1, Fig. 1b), of which 557 were selected for genetic analysis. BFT larvae were removed from all the right-side SB-90 and from the majority (75%) of the corresponding left-side SB-90 plankton samples. Larvae chosen for DNA extraction were all in preflexion to flexion developmental stages ranging in size from 2.6 to 9.9 mm SL and were estimated to be 4–11 days old. Larvae collected in 2016 were slightly smaller on average than those collected in 2017 (4.8 versus 5.5 mm respectively); 48% of the 2016 versus 74% of the 2017 larvae were greater than 5 mm in length.

**Table 1:** Sample (tow) identifier and capture location of larvae used in this study with inter and intra tow by relatedness category.

ID	Lon	Lat	No	DNA	Geno	Retain	Intra FSP	Intra HSP	Inter FSP	Inter HSP	Cross HSP
50654	− 90.4902	26.997	22	22	20	18	0	0	0	0	0
50679	− 92.0114	26.0094	20	19	10	5	0	0	0	0	0
50748	− 95.0126	26.0295	5	4	1	0	0	0	0	0	0
50837	− 93.0047	28.0148	47	21	7	0	0	0	0	0	0
50887	− 90.5046	27.5156	22	19	9	8	0	0	0	0	1
50907	− 89.5036	27.5139	47	40	19	6	0	0	0	0	0
50917	− 89.0093	28.0139	87	87	68	53	0	1	0	0	3
50952	− 88.006	27.9974	65	62	55	47	1	8	0	0	0
50972	− 88.0149	28.9977	27	22	10	7	0	0	0	0	0

D03755/6	− 87.767	26.1216	93	74	43	29	0	1	3	13	0
D03757/8	− 87.7733	26.1221	70	49	27	21	1	0	8	21	0
D03759/60	− 87.7773	26.1198	49	48	14	8	0	0	0	3	0
D03761/2	− 87.7796	26.1201	27	38	20	17	0	0	1	8	1
D03763/4	− 87.7926	26.1003	23	13	10	6	0	0	1	2	0
D03765/6	− 87.842	26.0626	14	10	6	5	0	0	0	5	0
D03767/8	− 87.8495	26.0536	43	39	33	25	0	0	3	11	1
D03769/70	− 87.8998	26.039	17	15	15	15	0	0	3	9	0
D03771/2	− 87.9148	26.0541	14	4	4	1	0	0	1	0	0
D03774	− 87.937	26.0328	1	0	0	0	–	–	–	–	–
D03775/6	− 87.956	26.0185	29	13	12	10	0	0	1	5	0
D03777/8	− 87.9551	26.0183	38	12	12	8	0	0	4	7	0
D03780	− 87.9478	25.9988	3	0	0	0	–	–	–	–	–
D03782	− 87.9555	25.9706	8	8	8	7	0	0	1	3	0
D03784	− 87.981	25.9295	7	1	1	1	0	0	1	1	0
D03785/6	− 88.0595	25.8776	27	14	11	8	0	0	1	6	0
D03787/8	− 88.0956	25.8675	31	10	9	6	0	0	1	6	0
D03789/90	− 88.1258	25.8593	31	15	1	1	0	0	0	1	0
D03791/2	− 88.1328	25.8438	146	82	77	74	1	7	8	37	2
D03793/4	− 88.1338	25.8215	31	5	5	4	0	0	0	4	0
D03795/6	− 88.1518	25.7866	6	6	6	5	0	0	0	2	0
D03797/8	− 88.1438	25.738	24	18	16	14	0	0	2	7	0
D03799/800	− 88.1835	25.6661	3	2	2	2	0	0	0	0	0
D03802	− 88.2126	25.6318	1	0	0	0	–	–	–	–	–
D03820	− 89.6234	25.5241	1	1	0	0	–	–	–	–	–
D03864	− 89.305	25.9336	1	0	0	0	–	–	–	–	–
D03987/8	− 88.6733	26.3316	40	21	5	4	0	0	0	0	0
D03989/90	− 88.6728	26.342	49	36	28	8	0	0	1	19	0
D03991/2	− 88.6631	26.365	35	18	4	1	0	0	0	0	0

The bold horizontal line demarcates the 2016 and 2017 sampling efforts.

ID, Tow identifier; Lon, Longitude; Lat, Latitude; No., The number of larvae sampled; DNA, The number of larvae from which DNA was extracted; Geno, The number of samples genotyped; Retain, The number of samples retained after data filtering; Intra FSP, Intra-sample full sibling pair; Intra HSP, Intra-sample half sibling pair; Inter FSP, Inter-sample full sibling pair; Inter HSP, Inter-sample half sibling pair; Cross HSP, Cross-cohort half sibling pair.

### Evaluation of larval DNA quality

DNA was successfully isolated from tail clips from 296 of the 342 larvae selected from 2016, and 552 of the 557 larvae selected from 2017 (Table 1). We recovered higher concentrations of DNA from the 2017 larvae, with 69% of samples above the target concentration of 30 ng/µL. DNA concentration increased rapidly with larval size; a high proportion of individuals with a minimum length of 6 mm SL had DNA concentrations above 30 ng/µL. A total of 568 larvae (199 larvae from 2016, and 369 from 2017) had adequate DNA concentrations for identification of single-nucleotide polymorphisms (SNPs) by high throughput genotyping-by-sequencing.

### DNA sequencing and quality control standards

A high proportion (93%) of the larvae submitted for sequencing were successfully genotyped using DArTseq™. Approximately 7% failed DArT sequencing quality standards and were eliminated from further processing. Four larvae in the 2017 sample were genetically identified as yellowfin tuna, (*Thunnus albacares*) and one as skipjack tuna (*Katsuwonus pelamis*); these were also eliminated. After all filtering steps (see “Methods” section), the final dataset used for sibship analysis contained 424 larvae; 144 representing seven out of nine different plankton sampling stations sampled in 2016, and 280 sub-sampled from 24 out of a total of 41 sampling stations in 2017.

In total, 66,319 unique binary SNP loci were identified. Prior to filtering, technical replicates indicated that 90.5% of loci were 100% reproducible across all samples and 96.5% of samples were 95% reproducible across all loci. Overall, 2.9% of loci contained complete data (were recovered across all individuals) and the level of missing loci per individual ranged from 15–40%. Forty-one percent of the original 66,319 SNPs identified were on the same sequenced fragment (i.e. secondary SNPs), and in these cases, a single SNP was randomly retained for sibship analysis. Allele frequencies were estimated for a final selected set of 6,641 loci, following additional filtering as described in “Methods” section.

### Identification of siblings and half siblings

**Figure 2:**
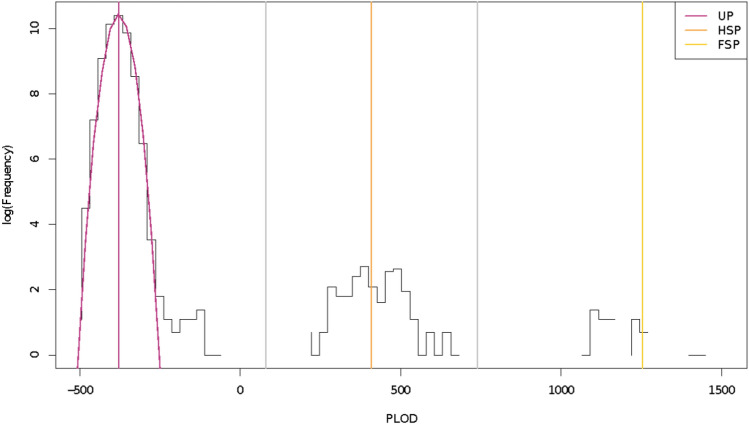
Psuedo-log-odds-ratios (PLOD) from the program "kinference" (Bravington et al., in prep) for discrimination of kinship pairs (half-siblings and full-siblings), on a vertical log scale. The vertical orange and yellow lines give the a priori expected mean PLOD for half-sibling pairs (HSP) and full-sibling pairs (FSP), respectively. The purple curve indicates the a priori expected distribution of unrelated pairs (UP), with its mean marked by the purple vertical line. The cutoffs between unrelated pairs, half-sibling pairs, and full-sibling pairs are denoted by gray vertical lines.

**Figure 3:**
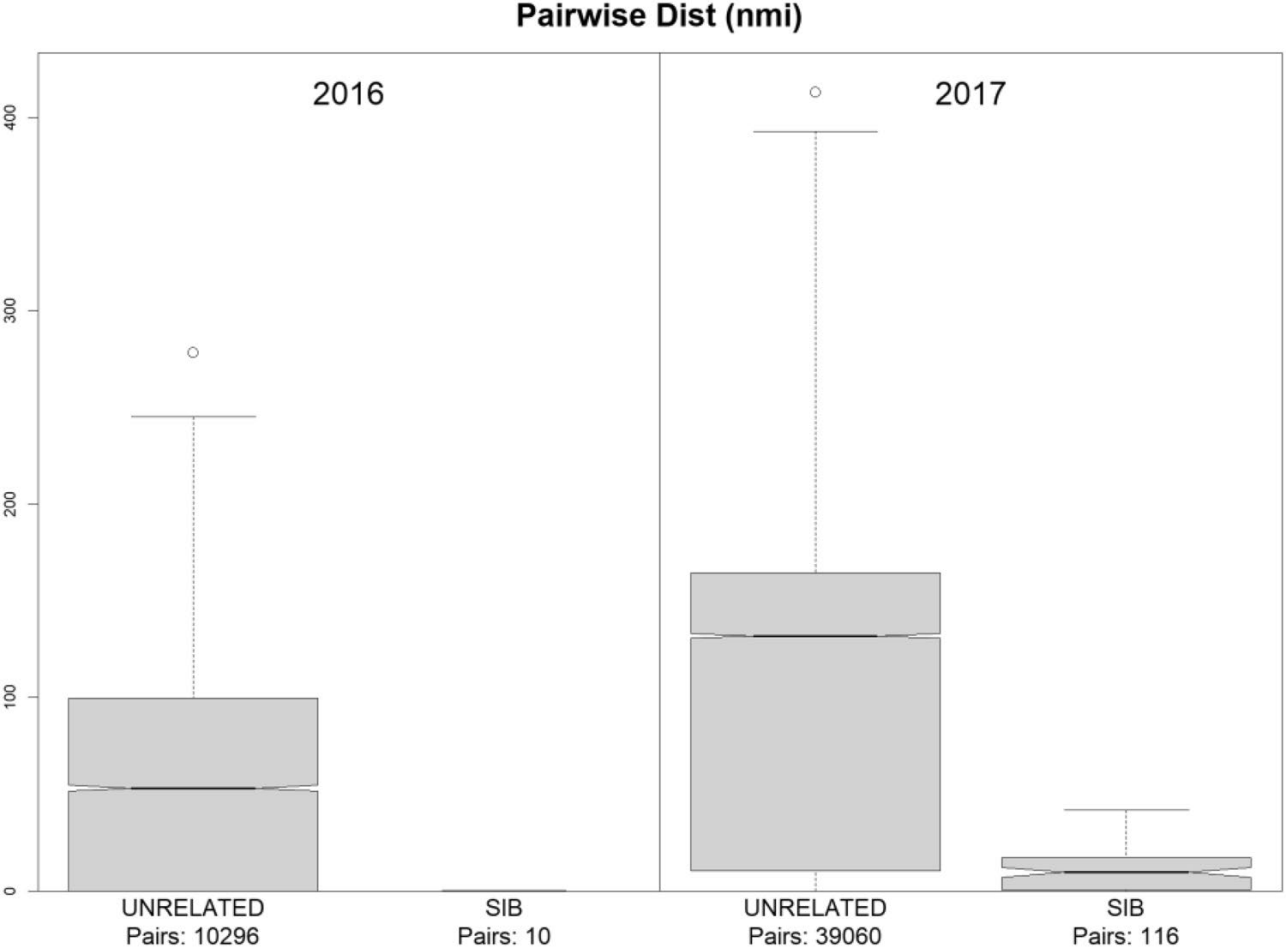
Intra-pair distances for sibling-pairs compared to unrelated pairs. Distances are based on tow locations; all 2016 sibling-pairs were within-tow, and therefore shown at zero distance.

We made pairwise comparisons of all larvae from both years to assign kinship of each pair to one of three categories: FSP, HSP, or UP (Unrelated Pair; all more-distant kinship categories). The comparison statistic was the log-ratio of the probabilities of each pair of larval genotypes (i) if the pair were half-siblings, versus (ii) if the pair were completely unrelated, which we refer to as Pseudo-Log-Odds (PLOD). The PLOD statistic gave clear separation between these three classes (see Fig. 2 and “Methods” section). Table 2 shows the overall pattern of sibship in the two years. Sibship was much higher in the 2017 targeted survey, with 56% of the samples having at least one sibling, compared to 12% in 2016. In both years, siblings were concentrated into certain tows. In 2016, out of the 7 tows with usable BFT larvae, just 2 tows contained within-tow siblings, and 0 tows had across-tow siblings. In 2017, out of the 24 tows with usable BFT larvae, 21 tows had within- or across-tow siblings, of which 5 had only within-tow siblings. Distances between sibling-pairs were much lower than between unrelated pairs (Fig. 3); there was no clear difference between full- and half-sibling pairs.

**Table 2:** Number of sampled larvae with and without half-sibs (HS) or full-sibs (FS).

Year	None	HS_no_FS	FS_and_HS	FS_no_HS	Total
2016	137	17	0	2	156
2017	140	140	15	22	317

Although we used only pairwise comparisons to identify siblings, many individuals had more than one sibling. There were some complex chains of sibship involving up to 7 individuals and 8 sibling pairs (Supplementary Material Tables S1, S2): for example, where A and B are HSP, so are A and C, but B and C are unrelated (because A shares a different parent with B than with C). Both full- and half-siblings were commonly found within the same chain. The pattern is consistent with small-group batch spawning. In 2017, some sibling-pairs were born almost 3 weeks apart, implying residency of spawners for at least that long. Perhaps surprisingly, one such pair was a FSP rather than a HSP; since a purely random re-mating with the same partner is extremely unlikely given such a large adult population, the mother and father must have stayed within the same spawning group for that time period.

To understand the implications of sibship for subsequent CKMR, the key step is to organize the pairs into Distinct Parent Groups (DPGs), each comprising all sampled offspring of one adult parent, which is of course unobserved and of unknown sex (see Supplementary Material C for an algorithm that does so, starting from pairwise sibships). The largest DPG comprised 5 larvae (Table 3), and the tabulation leads directly to the quantities required for evaluating CKMR implications, discussed next.

**Table 3:** Number of Distinct-Parent-Groups, *g*_*s*_, by size *s* (i.e., number of offspring in the group), plus summary statistics.

	1	2	3	4	5	≥ 6	${m_{J} }$	PotNP	NDP	VIF	${m_{{J{\text{eff}}}} }$
2016	291	9	1	0	0	0	156	312	301	1.08	144.9
2017	398	89	12	3	2	0	317	634	504	1.51	209.4

*m*_*J*_ is actual sample size; PotNP is *potential* number of distinct parents i.e. 2* mJ*, so ${{\text{PotNP}} = \sum s g_{s} }$; NDP is *actual* number of distinct parents, so ${{\text{NDP}} = \sum {g_{s} } }$; VIF is Variance-Inflation-Factor, i.e. the ratio of actual to ideal (as if independently-sampled) variance for a simplified POP-based CKMR abundance estimate, given by ${\sum {s^{{2}} } g_{s} /\sum s g_{s} }$; ${m_{{J{\text{eff}}}} }$ is the effective sample size, i.e. equivalent number of hypothetical independently-sampled juveniles that would yield the same variance as the actual sample. See “Methods” section and Supplementary Material B.

### Implications for CKMR modeling

The excess sibship observed in our larval sampling certainly breaks the effective independence of comparisons normally assumed in CKMR (as per the framework in^14^, although the framework is quite flexible and can be expanded to cover many situations). If we already know the POP result of comparing larva *i* to potential-parent adult *a*, then the fact that sibship is common amongst sampled larvae gives us predictive power for comparing any other larva *j* to *a*, because there is a substantial probability that *i* and *j* will be siblings and thus have the same relationship to *a.* That is true without needing to know whether *i* and *j* themselves are actually siblings. Importantly, though, there is nothing about our larval-sampling process (in conjunction with our adult-sampling, which takes place in a different place and in a different season) to suggest bias towards or against the offspring from any particular class of adults, in terms of larval per-capita sampling probability. Despite the patchy sampling, any single comparison between one sampled larva and one sampled adult should still have a probability of yielding a POP that is proportional to the annual fecundity of that adult, presumably as determined by its size. Thus, the basic conditions are still met for an unbiased size-based CKMR model, as per section 3.1.4 of^14^. Parameters would be estimated by maximizing the sum of log-likelihoods across pairwise comparisons, which ensures that the estimates are effectively unbiased by construction, even though that sum itself is not a true log-likelihood because of non-independence. Technically, the estimates are the solution of an Unbiased Estimating Equation (see section 4.1 of^14^, or Supplementary Material A).

Although bias is not a problem, the high sibship and loss of independence does affect the variance of CKMR estimates, because there is less information content than would come from an equal number of independently sampled (and therefore low-sibship) juveniles. That can be quantified in terms of an effective sample size, showing how many independently sampled juveniles would provide the same overall variance as our actual samples (Table 3); see “Methods” section. The effective-to-actual ratio is only slightly below 1 in 2016, but substantially so in 2017 when patches were deliberately re-targeted; nevertheless, effective sample size is still higher in 2017 than 2016 because the actual number of larvae was so much greater in 2017. Taking both years together, the combined effective sample size of 354 is about 75% of the combined actual size.

## Discussion

Our results show that GoM BFT larval survey samples can provide the crucial mark events for eventual CKMR estimates of adult abundance. The adult parents marked by larval samples can be directly recaptured in the fishery many years later as POPs, and indirectly through their progeny in future samples of larvae, as evidenced by the two cross-cohort HSPs (XHSPs) recovered in this study, which imply that a parent survived and spawned in the GoM in consecutive years. As more cohorts are sampled in future, the growing number of XHSPs could be used to estimate average adult survival rates, in addition to helping with the estimation of adult abundance^31^, as is now done for southern blue tuna^40^.

There is a modest level of sibship within our 2016 samples, and a high level (involving over half the samples) in 2017, but it turns out not to be high enough to cause serious problems for POP-based CKMR. High sibship per se does not lead to bias in CKMR by virtue of the statistical construction of the estimate, but it does increase variance, which can be summarized through a reduction in effective sample size. In a POP-based CKMR model, our effective sample size would be about 75% of nominal for the two years combined, or 66% of nominal for the targeted sampling of 2017. Since it is actually the product of adult and juvenile sample sizes which drives precision in CKMR^14^, one way to think about the 75% is that we will need about 33% more adult samples to achieve a given precision on abundance estimates than if we had somehow been able to collect the same number of “independent” juvenile samples (i.e. without oversampling siblings). That increase is appreciable but entirely achievable; for WBFT, it is logistically much easier to collect more feeding-ground adult samples than to collect more larvae, and at present there is no known practical way to collect large numbers of older, more dispersed, and thus more independent, juvenile western origin bluefin tuna (WBFT).

This study was motivated by the concern that sibship might be a serious impediment to use of WBFT larvae for CKMR. High levels of sibship have been found in larval collections for other taxa despite a pelagic larval phase, suggesting that abiotic factors can impede random mixing of larvae after a spawning event^41^. Our larval samples were only a few days old (4–11) and thus had little time to disperse since fertilization; our concern beforehand was that each tow might sample the offspring of a very small number of adults (one spawning group in one night), and in 2017 that repeatedly towing the same water mass might simply be resampling the same “family”. In practice, though, the cumulative effect was limited. Samples were not dominated by progeny from just a few adults; the maximum DPG size (i.e., number of offspring from any one adult) was 5, which is under 2% of the larval sample size. There are several possible reasons for this finding. First, plankton sample tows are typically standardized to a ten-minute duration, covering on average about 0.3 nautical miles. Based on continuous plankton cameras^42^, each tow is likely to tow through multiple patches of zooplankton, and therefore potentially multiple patches of BFT larvae. Second, spawning aggregations of BFT may contain many adults. For example, on the spawning grounds near the Balearic Islands in the Mediterranean, purse seine fisheries target spawning fish and individual net sets routinely capture upwards of 500 mature individuals^43^. These numbers suggest that BFT spawner aggregations can be quite large, although the number of individuals that contribute gametes to a single spawning event may be lower. The results of this study pose intriguing scenarios for understanding BFT larval ecology and spawning behavior, which could be explored with larger sample sizes paired with data on oceanographic conditions, direct observation of spawning aggregations, and modeling to compare observed and predicted dispersal. The results of this study are based on just two years of sampling, and numerous practical and theoretical challenges remain to fully understand BFT reproduction in the GoM.

Our sibship impact calculations assume use of an unmodified adult-size-based CKMR POP model, where each juvenile is compared to each adult taking into account the latter's size (e.g.,^14^). That will give unbiased estimates, which we regard as essential in a CKMR model. However, for WBFT the estimates are not fully statistically efficient*,* in that some adults receive more statistical weight than others because they are marked more often (by having a large DPG), and thus variance might not be the lowest achievable. Modifying the model to fix that would be simple in a “cartoon” CKMR setting where all adults are identical (e.g., Fig. 1 of^14^), simply by first condensing each DPG to a single representative, then only using those representatives (rather than all the larvae) in POP comparisons. Each marked parent then receives the same weight, giving maximum efficiency. For the cartoon, this condensed-DPG model still gives an unbiased estimate of abundance, because each DPG has one parent of given sex, and the chance of any sampled cartoon adult of that sex being that parent is 1/*N*. The DPG-condensed effective sample size is simply half the number of distinct parents, which would be a little larger than the effective sample sizes for the unmodified model shown in Table 3; e.g., in 2017, 504/2 = 252 versus 209. However, no such straightforward improvement is available for an adult-size-based CKMR model such as is needed for WABFT. Using condensed DPGs directly would bias the juvenile sampling against larger more-fecund adults, whose DPGs will tend on average to be larger and thus to experience disproportionate condensation. Those adults would be marked less often by the DPG-condensed juveniles than the model assumes, violating the basic requirements for unbiased CKMR in^14^. A more sophisticated model might be able to combine unbiasedness with higher efficiency but, since the unmodified adult-size-based POP model that we expect to use is unbiased and only mildly inefficient (at worst 209/252 = 83% efficient, in 2017) there seems no particular need for extra complications at present. However, that may not hold true if we eventually move to a POP + XHSP model, where the impact on unmodified CKMR variance is worse (though there is still no bias, for the same reason as with POPs). Intuitively, the biggest impact that a DPG of size 5 can have in a POP model is to suddenly raise the number of POPs by 5 if its parent happens to be sampled; within a useful total of, say, 75 POPs, the influence is not that large. But if two DPGs both of size 5 in different cohorts happen to share a parent, then the total of XHSPs suddenly jumps by 25— likely a substantial proportion of total XHSPs. Supplementary Material B also includes effective sample size formulae for a simplified XHSP-only model, which demonstrate the increased impact of within-cohort sibship; for our WBFT samples, it turns out that the XHSP-effective size is slightly lower for the targeted 2017 samples (110) than for the 2016 samples (130), unlike the POP-only effective size. Dropping from a maximum theoretical effective sample size of 252 (half the number of DPGs) down to 110 would be rather inefficient and would increase the number of years of sampling required to yield a useful XHSP dataset. This motivates developing a modified POP + XHSP model that retains unbiasedness without sacrificing too much efficiency. In principle, that can be done by condensing each DPG but then conditioning its comparison probabilities on the DPG’s original size, in accordance with the framework in^14^. This is a topic for subsequent research, and the results will inform future sampling strategy decisions for WBFT.

One potential difficulty for western BFT CKMR might occur if a substantial proportion of animals reaching maturity are the offspring of “Western” (in genetic terms) adults who persistently spawn in the western North Atlantic but outside the GoM. However, as long as the adults marked by GoM larvae are well mixed at the time of sampling with any western adults that do spawn outside of the GoM, the total POP-based population estimate of genetically-western BFT from CKMR will remain unbiased. Given evidence from tagging of widespread adult movements within the western North Atlantic^2^, good mixing in the sampled feeding grounds seems likely; so, even if successful non-GoM western BFT spawning really is commonplace, there should not be a problem with relying on GoM larvae for at least the POP component of CKMR^14^.

Studies of fish early life history have long been considered to have great potential to provide novel insight into the unique population dynamics of fishes^44–46^. Sampling efforts aimed at estimating fish recruitment dynamics have spawned a diversity of larval survey programs. Examples of these long-term programs include the California Cooperative Oceanic Fisheries Investigations, International Council for the Exploration of the Sea (ICES) surveys in the North Atlantic and adjacent areas, Southeast Monitoring and Assessment Program (SEAMAP) in the GoM, Ecosystem Monitoring (EcoMon) in the Northeast U.S., and numerous others, many of which provide indices of larval abundance widely used in fisheries and ecosystem assessments. Yet, as a result of the inherent patchiness of larvae^42^, sampling variability, and highly variable density dependent mortality^45^, fisheries scientists have often struggled to determine how larval surveys relate to the adult fish populations. Inclusion of estimates of sibship among larvae collected in surveys could refine estimates of adult spawning stock biomass estimated from these surveys.

The results of this study also represent products of decades of work and coordination in obtaining high-quality DNA from larval specimens. Key steps to successful genotyping of larvae include ensuring that larvae are preserved, sorted, and handled in 95% non-denatured ethanol. In addition, strict instrument cleaning protocols must be followed, and stomachs should be removed or avoided (this study used larval tails and, when possible, eyes to avoid cross contamination of prey contents, including possible congeners and other BFT individuals). Exposure to hot lamps during the sorting and dissection processes should also be minimized to ensure that DNA quality is sufficiently high for genotyping-by-sequencing. Although the tissues available for genetic analysis were limited by the needs of other experiments that required BFT tissues, otoliths, gut contents, and other information from the same larvae, we were able to successfully genotype most larvae greater than 6 mm SL and identify thousands of informative SNPs. The lower size limit of larvae could likely be decreased if whole specimens were available for genotyping, although the use of younger larvae could increase the incidence of sibship.

In summary, while we observed both FSPs and HSPs in larval collections, with elevated sibship overall and with siblings being more prevalent within tows and in nearby tows, the level of sibship was sufficiently low that collections of GoM BFT larvae can still provide the critical genetic mark of parental genotypes required for CKMR. Our results demonstrate a crucial proof of concept and are the first step towards an operational CKMR modelling estimate of spawning stock abundance for western BFT.

## Methods

BFT larvae were collected during two oceanographic surveys in the northern GoM. In 2016, BFT larvae were collected as part of the SEAMAP standard ichthyoplankton survey (Fig. 1a), and in 2017 they were collected during a targeted study on the trophic dynamics of BFT^44,45^ (Fig. 1b). Larvae from 2016 were collected aboard the NOAA Ship *Oregon II* using a bongo net (61 cm diameter) fitted with 505 µm mesh paired nets aligned adjacently on the right and left sides of the tow cable and pulled in an undulating pattern between the surface and 10 m for 10 min, (SB-60 net henceforth). The larval survey follows a grid that is spaced approximately at 30 nm intervals during the peak of BFT spawning season from 24° N to 30° N and 83.5° W to 96° W^47^. A total of 118 stations were completed and BFT larvae were counted from the right SB-60 plankton sample and were designated for standard SEAMAP survey protocols. The corresponding left SB-60 plankton samples were retained exclusively for genetics, and tows with the highest number of BFT larvae were sub-sampled.

In 2017, larvae were collected aboard the NOAA Ship *Nancy Foster* using a bongo net (90 cm diameter) fitted with 505 µm mesh and towed in an undulating pattern between the surface and 25 m for approximately 10 min (SB-90 net henceforth)^48^. The primary objective of the targeted survey was to locate and intensively sample larval BFT. A Lagrangian buoy with a satellite tracker was released and a three-to-four-day experiment commenced once a minimum of five BFT larvae were detected in the plankton sample. The ship remained in close proximity (within 2 nm) to the buoy for the two-week duration of the Lagrangian experiments.

A mechanical flowmeter (2030 General Oceanics) recorded the volume of water filtered by all bongo nets. All collections were preserved in 95% ethanol and sorted for BFT larvae at the Southeast Fisheries Science Center (SEFSC) in Miami, Florida. Larvae were identified morphologically, placed in individual vials, and standard length (SL) was measured to the nearest hundredth of a millimeter using a Leica M205C dissecting microscope, a digital camera, and Leica Application Suite (Leica Microsystems) software (Leica, Wetzlar, Germany). Larval age (in number of days) was estimated from SL using a published growth curve^49^ and assigned a developmental stage: preflexion, flexion or postflexion^50^. Larvae from the nine most BFT abundant stations were subsampled for genetic analysis from the 2016 collection (n = 342), while 557 larvae were subsampled from the 2017 collection.

Larvae selected for DNA extractions were in preflexion to postflexion developmental stages during both surveys with body sizes that ranged from 2.6 to 9.9 mm SL. A small tail clip and, when possible, an eye was excised and placed into individual vials of 95% ethanol. Dissection utensils were cleaned in distilled water followed by 95% ethanol and sterilized over a Bunsen burner between each specimen to avoid cross-contamination. For the 2016 collection, tail clips and eyes were subsampled at SEFSC while whole specimens from the 2017 collection were sent to the Virginia Institute of Marine Science (VIMS) for subsampling following the same procedures outlined above and stored at − 20 °C until DNA was isolated. Prior to DNA isolation, larval tissues were placed in 90 µL of lysis buffer (25 M Tris, 25 mM EDTA, 2 M GuHCl, 5 mM CaCl2, 0.5% Triton-X-100, 1% N-Lauroyl-Sarcosine) that included 20 µL of 20 mg/mL Proteinase K and incubated overnight at 55 °C. Following tissue digestion, DNA was isolated following a standard magnetic bead protocol using carboxylated magnetic beads (MCLAB, San Francisco, CA). DNA bound to the beads was washed in 70% ethanol twice before being eluted in 0.1X TE. DNA was quantified using a NanoDrop spectrophotometer (ThermoFisher Scientific, Waltham, MA) and a subset of DNA isolations were visualized on 1% agarose gels to ensure that high molecular weight DNA was recovered. Samples with sufficient DNA quality and quantity (30 ng/µL of high molecular weight DNA) were sent to Diversity Arrays Technology Pty. Ltd. (DArT PL; Canberra, Australia) for DArTseq™ 1.0^13^ genotyping by sequencing of a reduced representation genomic libraries^51^. Preliminary quality filtering and SNP identification was completed at Diversity Arrays using a proprietary analytical software pipeline, DArTsoft14.

The larval dataset was further filtered and analyzed, first using DArTseq cluster counts output from the DArT-Soft14 proprietary genotyping pipeline, then using the R package “kinference” of Bravington et al., (*in prep.*). Briefly, uninformative loci (minor allele frequency, MAF, < 0.05), polyploid loci, and loci with a mean read-depth under 25 were removed. For purposes of allele frequency estimation, we temporarily excluded individuals with mean observed heterozygosity levels greater than upper quartiles (indicating potential cross contamination) or less than lower quartiles (indicating potential poor quality DNA). After estimating allele frequencies on the reduced set of individuals, we re-incorporated the exclusions and then further filtered the dataset to remove inadvertently duplicated samples. Individuals with outlying genotype likelihoods based on allele frequencies (n = 31) were discarded, as were individuals with outlying elevated observed heterozygosity relative to HWE (potentially indicating contaminated samples, n = 13) and those with outlying low observed heterozygosity relative to HWE (potentially indicating sample degradation, n = 18). Finally, pairwise kin-finding was performed using the R package “kinference”. The statistic used for each pairwise comparison was the log-ratio of the probability of the pair’s genotypes if the pair was truly a HSP compared to the probability if the pair was completely unrelated (UP), which we refer to as Pseudo-Log-Odds (PLOD)^14,52^. This gave clear discrimination between the three categories, including separation of FSPs from HSPs. Although there are other second-order kin which are genetically indistinguishable from HSPs, they should not be an issue here. Larvae-only datasets cannot contain any grandparent-grandchild pairs that would mimic HSPs genetically^14,30^. Full-Thiatic Pairs, i.e. an individual and the offspring of its full sibling (e.g., aunt-niece; “thiatic” denoting aunt or uncle), which also have the same relatedness as HSPs, should be demographically rare in same-cohort comparisons because of the generation gap between pair members, and because surviving full-siblings (as opposed to half-siblings) should be uncommon amongst adult BFT. For the former reason, larvae-only datasets collected over a small number of years are also unlikely to contain many Half-Thiatic Pairs (an individual and the offspring of its half-sibling) that can overlap with the left-hand side of the HSP PLOD distribution. Consequently, PLOD separation between the three kin classes FSP, HSP, UP was very clear for this dataset, so kinships were based on simple segmentation of the PLOD axis (Fig. 2).

The unbiased nature of CKMR estimates even in the presence of larval sibship can be shown mathematically, as in Supplementary Material A; it follows directly from using the sum of pairwise log-likelihoods as the basis for estimation. There is an impact on variance, though, and to evaluate that, we derived analytical formulae for the simple “cartoon” CKMR model with a single juvenile cohort and where all adults are equivalent (e.g. Fig. 1 of^14^); see Supplementary Material B. Strictly speaking, those formulae do not apply exactly to WBFT because, in order to avoid bias, we will need to use a more complicated CKMR model involving individual adult sizes; and large adults (with larger DPGs, on average) will be somewhat differently affected by larval sibship than small adults. Nevertheless, the point of the formulae is to show the increase of the variance in number of kin-pairs found under high sibship, which should be similar in the simple and complicated models. Variance in parameter estimates is directly driven by the number of kin-pairs, so we expect the inflated-variance results to also be broadly applicable to more complicated models such as for WBFT, where there are no closed-form parameter estimates and where no comparable simple variance formulae exist. The resulting formula for “effective sample size” exhibits a quadratic (and therefore not completely obvious—e.g., not just based on the number of distinct parents) dependence on the pattern of sibship shown in the DPG tabulation of Table 3.

### Approval for animal experiments

The Regional Administrator of NOAA Fisheries Service’s Southeast Regional Office (SERO) authorized collection of ichthyoplankton samples in the Economic Exclusive Zone (EEZ) of the Gulf of Mexico on Southeast Area Monitoring and Assessment Program (SEAMAP) surveys as part of a Scientific Research Permit (SRP). In addition, part of the 2017 survey was conducted in Mexico with a fishing permit (PPFE/DGOPA-018/17) from the Comisión Nacional de Acuacultura y Pesca. The methods used to collect biological data in this study are considered scientific research in accordance with 50 CFR 600.10 and 600.745 and are not subject to fishing regulations or essential fish habitat requirements.

### Ethics declarations

The authors have complied with ARRIVE guidelines.

## Supplementary Information

Supplementary Information 1.

Supplementary Information 2.

## Data Availability

The datasets generated during and/or analyzed during the current study are available from the corresponding author on reasonable request.
