# Supplementary material for: Low levels of sibship encourage use of larvae in western Atlantic bluefin tuna abundance estimation by close-kin mark-recapture
Source: Sci Rep. 2022 Nov 3;12:18606. doi: 10.1038/s41598-022-20862-9 (PMC9633702; doi:10.1038/s41598-022-20862-9)
Supplement: Supplementary file 1 — Supplementary Information 1. [file 41598_2022_20862_MOESM1_ESM.docx]

# Supplement

## D: Tabulations of sib-pairs

**Table S1.** Table of full sibling pairs. Shading separates sibling groups. Asterisks mark individuals appearing in both full and half sibling results. Colors separate related groups. SL = standard length.

|  |  |  |  |  |  | |  |  |  |  |
| --- | --- | --- | --- | --- | --- | --- | --- | --- | --- | --- |
| FULL SIBLINGS | |  |  |  |  | |  |  |  |  |
| Cohort | Related Pair | | Age & SL (days. mm) | | | | | PLOD | Tow Distance (nm) | Time Apart (days) |
|  | Sample 1 | Sample 2 | Sample 1 | | | Sample 2 | |  |  |  |
| 2016 | OR3170381 | OR3170372 | 7 | 4.73 | | 7 | 4.62 | 1223.36 | 0 | 0 |
| 2017 | N17040045* | N17040044* | 8 | 5.44 | | 7 | 4.86 | 1267.30 | 0 | 0 |
|  | N17040200* | N17040044* | 6 | 4.4 | | 7 | 4.86 | 1481.06 | 11.62 | 1 |
|  | N17040200* | N17040045* | 6 | 4.4 | | 8 | 5.44 | 1063.93 | 11.62 | 1 |
|  | N17040347* | N17040044* | 6 | 4.27 | | 7 | 4.86 | 1253.62 | 5.81 | 1 |
|  | N17040347* | N17040045* | 6 | 4.27 | | 8 | 5.44 | 1307.53 | 5.81 | 1 |
|  | N17040347* | N17040200* | 6 | 4.27 | | 6 | 4.4 | 1099.45 | 6.08 | 0 |
|  | N17040223* | N17040043* | 7 | 4.81 | | 10 | 6.19 | 1231.81 | 0.34 | 0 |
|  | N17040318* | N17040048* | 5 | 3.92 | | 9 | 5.8 | 1408.12 | 5.81 | 1 |
|  | N17040482* | N17040048* | 5 | 3.86 | | 9 | 5.8 | 1125.08 | 25.59 | 2 |
|  | N17040482* | N17040318* | 5 | 3.86 | | 5 | 3.92 | 1102.50 | 19.81 | 1 |
|  | N17040353 | N17040220 | 7 | 4.98 | | 6 | 4.39 | 1380.59 | 8.95 | 1 |
|  | N17040412 | N17040051 | 7 | 4.65 | | 9 | 5.55 | 1237.39 | 21.29 | 2 |
|  | N17040419 | N17040317 | 6 | 4.15 | | 8 | 5.12 | 1195.08 | 17.35 | 1 |
|  | N17040476 | N17040216 | 5 | 3.85 | | 6 | 4.37 | 1130.81 | 12.23 | 0 |
|  | N17040484 | N17040393 | 6 | 4.19 | | 6 | 4.31 | 1113.35 | 14.19 | 1 |
|  | N17040539 | N17040474 | 11 | 6.42 | | 6 | 4.24 | 1164.71 | 0 | 0 |
|  | N17040580 | N17040297 | 11 | 6.58 | | 6 | 4.28 | 1146.99 | 0 | 0 |
|  | N17040669 | N17040003 | 6 | 4.5 | | 4 | 3.54 | 1427.66 | 0.69 | 0 |
|  | N17040685* | N17040366* | 8 | 5.1 | | 7 | 4.68 | 1139.04 | 10.09 | 1 |
|  | N17040722* | N17040465* | 6 | 4.49 | | 6 | 4.21 | 1108.16 | 9.68 | 0 |
|  | N17040748 | N17040259 | 8 | 5.43 | | 7 | 4.78 | 1083.04 | 0 | 0 |
|  | N17040764* | N17040527* | 7 | 4.89 | | 7 | 4.94 | 1160.74 | 41.70 | 18 |
|  | N17040230 | N17040167 | 9 | 5.78 | | 9 | 5.75 | 850.41 | 6.03 | 1 |
| Average (Range) | | | 4.76 (3.54–6.58) | | | | | 1195.91 (850.41–1481.06) | 9.78 (0–41.70) | 1.38 (0–18) |
|  |  |  |  |  |  | |  |  |  |  |

**Table S2.** Table of half sibling pairs. Shading separates sibling groups. Asterisks mark individuals appearing in both full and half sibling results. Colors separate related groups. SL = standard length.

| HALF SIBLINGS | | |  |  |  |  |  |  |  |  |
| --- | --- | --- | --- | --- | --- | --- | --- | --- | --- | --- |
| Cohort | Related Pair | | Age & SL (mm) | | | | PLOD | Tow Distance (nm) | Time Apart (days) |  |
|  | Sample 1 | Sample 2 | Sample 1 | | Sample 2 | |  |  |  |  |
| 2016 | OR3170275 | OR3170262 | 9 | 5.58 | 9 | 5.86 | 419.05 | 0 | 0 |  |
|  | OR3170345 | OR3170341 | 6 | 4.33 | 10 | 6 | 498.84 | 0 | 0 |  |
|  | OR3170368 | OR3170328 | 6 | 4.31 | 7 | 4.83 | 354.67 | 0 | 0 |  |
|  | OR3170376 | OR3170328 | 6 | 4.35 | 7 | 4.83 | 331.44 | 0 | 0 |  |
|  | OR3170376 | OR3170368 | 6 | 4.35 | 6 | 4.31 | 500.57 | 0 | 0 |  |
|  | OR3170382 | OR3170334 | 6 | 4.25 | 9 | 5.64 | 282.66 | 0 | 0 |  |
|  | OR3170327 | OR3170335 | 8 | 5.03 | 7 | 4.75 | 447.97 | 0 | 0 |  |
|  | OR3170330 | OR3170353 | 6 | 4.49 | 10 | 5.96 | 199.68 | 0 | 0 |  |
|  | OR3170342 | OR3170354 | 6 | 4.48 | 9 | 5.7 | 486.87 | 0 | 0 |  |
| 2017 | N17040042 | N17040013 | 7 | 4.86 | 4 | 3.53 | 495.65 | 0.38 | 0 |  |
|  | N17040175 | N17040171 | 6 | 4.34 | 5 | 3.73 | 501.81 | 3.02 | 0 |  |
|  | N17040569 | N17040171 | 5 | 3.79 | 5 | 3.73 | 591.77 | 17.67 | 2 |  |
|  | N17040569 | N17040175 | 5 | 3.79 | 6 | 4.34 | 542.51 | 14.65 | 2 |  |
|  | N17040186 | N17040046 | 8 | 5.28 | 7 | 4.85 | 413.05 | 10.96 | 1 |  |
|  | N17040385 | N17040241 | 9 | 5.89 | 7 | 4.84 | 456.43 | 11.29 | 1 |  |
|  | N17040814 | N17040046 | 8 | 5.1 | 7 | 4.85 | 453.42 | 17.18 | 2 |  |
|  | N17040814 | N17040186 | 8 | 5.1 | 8 | 5.28 | 272.47 | 6.22 | 1 |  |
|  | N17040814 | N17040241 | 8 | 5.1 | 7 | 4.84 | 466.76 | 17.56 | 2 |  |
|  | N17040214 | N17040091 | 6 | 4.38 | 8 | 5.13 | 252.74 | 10.7 | 2 |  |
|  | N17040219 | N17040043* | 5 | 3.97 | 10 | 6.19 | 285.73 | 0.38 | 0 |  |
|  | N17040223* | N17040219 | 7 | 4.81 | 5 | 3.97 | 452.01 | 0 | 0 |  |
|  | N17040304 | N17040043* | 7 | 4.73 | 10 | 6.19 | 301.88 | 4.12 | 1 |  |
|  | N17040304 | N17040223* | 7 | 4.73 | 7 | 4.81 | 491.91 | 4.5 | 1 |  |
|  | N17040269 | N17040086 | 6 | 4.42 | 8 | 5.33 | 484.35 | 21.34 | 2 |  |
|  | N17040271 | N17040268 | 7 | 4.74 | 6 | 4.4 | 382.28 | 0 | 0 |  |
|  | N17040310 | N17040067 | 5 | 3.64 | 10 | 6.26 | 401.88 | 4.57 | 1 |  |
|  | N17040334 | N17040067 | 6 | 4.32 | 10 | 6.26 | 330.95 | 4.57 | 1 |  |
|  | N17040323 | N17040260 | 7 | 4.83 | 7 | 4.77 | 234.78 | 17 | 1 |  |
|  | N17040329 | N17040321 | 5 | 3.89 | 9 | 5.67 | 371.73 | 0 | 0 |  |
|  | N17040335 | N17040290 | 7 | 5.01 | 5 | 3.98 | 547.42 | 18.15 | 2 |  |
|  | N17040343 | N17040267 | 5 | 3.94 | 7 | 4.74 | 587.51 | 13.99 | 1 |  |
|  | N17040489 | N17040267 | 9 | 5.92 | 7 | 4.74 | 409.79 | 0 | 0 |  |
|  | N17040489 | N17040343 | 9 | 5.92 | 5 | 3.94 | 465.15 | 13.99 | 1 |  |
|  | N17040546 | N17040267 | 8 | 4.55 | 7 | 4.74 | 508.15 | 0 | 0 |  |
|  | N17040546 | N17040343 | 8 | 4.55 | 5 | 3.94 | 317.5 | 13.99 | 1 |  |
|  | N17040546 | N17040489 | 8 | 4.55 | 9 | 5.92 | 387.71 | 0 | 0 |  |
|  | N17040562 | N17040267 | 9 | 5.7 | 7 | 4.74 | 374.32 | 0 | 0 |  |
|  | N17040371 | N17040120 | 5 | 3.9 | 9 | 5.8 | 339.32 | 10.72 | 1 |  |
|  | N17040377 | N17040159 | 8 | 5.36 | 6 | 4.36 | 287.2 | 6.84 | 0 |  |
|  | N17040387 | N17040366* | 5 | 3.91 | 7 | 4.68 | 516.7 | 0.05 | 0 |  |
|  | N17040405 | N17040215 | 7 | 4.66 | 5 | 4.02 | 463.57 | 6.24 | 0 |  |
|  | N17040406 | N17040166 | 8 | 5.26 | 8 | 5.08 | 398.04 | 12.61 | 1 |  |
|  | N17040411 | N17040174 | 6 | 4.26 | 5 | 4.04 | 458.05 | 9.59 | 1 |  |
|  | N17040421 | N17040201 | 7 | 4.96 | 7 | 4.82 | 382.78 | 8.44 | 1 |  |
|  | N17040460 | N17040254 | 5 | 3.87 | 7 | 4.56 | 340.17 | 21.96 | 2 |  |
|  | N17040461 | N17040036 | 6 | 4.21 | 9 | 5.71 | 385.68 | 21.58 | 2 |  |
|  | N17040465* | N17040047 | 6 | 4.21 | 9 | 5.91 | 359.57 | 21.58 | 2 |  |
|  | N17040722* | N17040047 | 6 | 4.49 | 9 | 5.91 | 357.56 | 12.47 | 2 |  |
|  | N17040808 | N17040044* | 6 | 4.48 | 7 | 4.86 | 370.36 | 0 | 0 |  |
|  | N17040808 | N17040200* | 6 | 4.48 | 6 | 4.4 | 285.11 | 10.91 | 1 |  |
|  | N17040808 | N17040347* | 6 | 4.48 | 6 | 4.27 | 427.95 | 7.59 | 1 |  |
|  | N17040816 | N17040044* | 6 | 4.46 | 7 | 4.86 | 324.61 | 17.18 | 2 |  |
|  | N17040816 | N17040045* | 6 | 4.46 | 8 | 5.44 | 517.21 | 17.18 | 2 |  |
|  | N17040816 | N17040047 | 6 | 4.46 | 9 | 5.91 | 364.68 | 17.18 | 2 |  |
|  | N17040816 | N17040200* | 6 | 4.46 | 6 | 4.4 | 408.93 | 6.27 | 1 |  |
|  | N17040477 | N17040242 | 10 | 5.95 | 7 | 4.72 | 462.54 | 21.96 | 2 |  |
|  | N17040488 | N17040242 | 5 | 3.84 | 7 | 4.72 | 390.28 | 21.96 | 2 |  |
|  | N17040488 | N17040477 | 5 | 3.84 | 10 | 5.95 | 487.8 | 0 | 0 |  |
|  | N17040483 | N17040481 | 5 | 3.88 | 5 | 3.82 | 391.24 | 0 | 0 |  |
|  | N17040570 | N17040481 | 4 | 3.57 | 5 | 3.82 | 279.31 | 0.69 | 1 |  |
|  | N17040570 | N17040483 | 4 | 3.57 | 5 | 3.88 | 368.1 | 0.69 | 1 |  |
|  | N17040664 | N17040481 | 6 | 4.52 | 5 | 3.82 | 514.36 | 21.2 | 2 |  |
|  | N17040664 | N17040483 | 6 | 4.52 | 5 | 3.88 | 536.61 | 21.2 | 2 |  |
|  | N17040664 | N17040570 | 6 | 4.52 | 4 | 3.57 | 398.74 | 21.87 | 3 |  |
|  | N17040486 | N17040325 | 6 | 4.26 | 8 | 5.07 | 389.62 | 17 | 1 |  |
|  | N17040492 | N17040472 | 7 | 4.62 | 6 | 4.22 | 418.93 | 0 | 0 |  |
|  | N17040498 | N17040212 | 10 | 6.39 | 8 | 5.06 | 488.95 | 10.64 | 0 |  |
|  | N17040499 | N17040495 | 6 | 4.23 | 12 | 7.13 | 386.49 | 0 | 0 |  |
|  | N17040503 | N17040405 | 7 | 4.96 | 7 | 4.66 | 391.69 | 4.4 | 0 |  |
|  | N17040510 | N17040264 | 8 | 5.34 | 7 | 4.76 | 467.09 | 0 | 0 |  |
|  | N17040527* | N17040305 | 7 | 4.94 | 6 | 4.31 | 396.77 | 17.45 | 1 |  |
|  | N17040764* | N17040305 | 7 | 4.89 | 6 | 4.31 | 481.98 | 17.03 | 19 |  |
|  | N17040533 | N17040462 | 7 | 4.56 | 5 | 3.83 | 525.4 | 0 | 0 |  |
|  | N17040544 | N17040526 | 7 | 4.94 | 7 | 4.58 | 477.76 | 0 | 0 |  |
|  | N17040547 | N17040229 | 6 | 4.55 | 8 | 5.04 | 296.35 | 21.96 | 2 |  |
|  | N17040555 | N17040293 | 6 | 4.53 | 5 | 3.67 | 468.28 | 1.08 | 1 |  |
|  | N17040583 | N17040349 | 4 | 3.6 | 7 | 4.69 | 197.07 | 14.65 | 2 |  |
|  | N17040659 | N17040053 | 6 | 4.14 | 8 | 5.18 | 459.59 | 0.38 | 0 |  |
|  | N17040665 | N17040502 | 6 | 4.51 | 7 | 4.62 | 317.08 | 21.2 | 2 |  |
|  | N17040665 | N17040513 | 6 | 4.51 | 5 | 3.81 | 392.95 | 21.2 | 2 |  |
|  | N17040768 | N17040502 | 10 | 6.15 | 7 | 4.62 | 392.53 | 0.42 | 18 |  |
|  | N17040768 | N17040665 | 10 | 6.15 | 6 | 4.51 | 479.23 | 20.78 | 20 |  |
|  | N17040671 | N17040521 | 8 | 5.08 | 7 | 4.59 | 350.76 | 21.2 | 2 |  |
|  | N17040684 | N17040407 | 7 | 4.93 | 4 | 3.62 | 297.76 | 16.02 | 2 |  |
|  | N17040685* | N17040387 | 8 | 5.1 | 5 | 3.91 | 360.14 | 9.75 | 1 |  |
|  | N17040694 | N17040041 | 6 | 4.11 | 10 | 6.14 | 444.46 | 0.38 | 0 |  |
|  | N17040714 | N17040554 | 7 | 4.91 | 6 | 4.54 | 413.81 | 21.64 | 2 |  |
|  | N17040717 | N17040532 | 7 | 4.9 | 7 | 4.57 | 561.09 | 21.58 | 2 |  |
|  | N17040721 | N17040221 | 5 | 4.07 | 5 | 4.02 | 443.49 | 0.38 | 0 |  |
|  | N17040728 | N17040011 | 8 | 5.37 | 4 | 3.53 | 295.89 | 19.72 | 2 |  |
|  | N17040728 | N17040048* | 8 | 5.37 | 9 | 5.8 | 405.67 | 19.35 | 2 |  |
|  | N17040728 | N17040318* | 8 | 5.37 | 5 | 3.92 | 508.87 | 14.77 | 1 |  |
|  | N17040728 | N17040482* | 6 | 5.37 | 5 | 3.86 | 492.18 | 2.23 | 0 |  |
|  | N17040729 | N17040566 | 6 | 4.12 | 8 | 5.42 | 304.37 | 2.29 | 0 |  |
|  | N17040742 | N17040037 | 5 | 4.07 | 11 | 6.53 | 486.82 | 21.16 | 2 |  |
|  | N17040745 | N17040693 | 10 | 6.13 | 7 | 4.91 | 314.72 | 21.96 | 2 |  |
|  | N17040770 | N17040404 | 10 | 6.12 | 5 | 3.82 | 427.43 | 3.98 | 18 |  |
|  | N17040810 | N17040385 | 8 | 5.13 | 9 | 5.89 | 310.99 | 6.27 | 1 |  |
|  | N17040816 | N17040347* | 6 | 4.46 | 6 | 4.27 | 502.98 | 9.59 | 1 |  |
|  | N17040819 | N17040295 | 5 | 3.78 | 5 | 4.01 | 377.72 | 5.07 | 1 |  |
|  | N17040821 | N17040262 | 8 | 5.09 | 8 | 5.4 | 336.19 | 0 | 0 |  |
|  | N17040822 | N17040744 | 7 | 4.88 | 5 | 4.09 | 368.68 | 0 | 0 |  |
| Cross | N17040540 | OR3170261 | F | 3.55 | F | 6.55 | 463.11 | 138.08 | 349 |  |
|  | N17040544 | OR3170244 | PRF | 4.94 | F | 6.19 | 460.13 | 138.08 | 349 |  |
| Average (Range) | | | 4.73 (3.53–7.13) | | | | 408.41 (197.07–591.77) | 15.11 (0–138.08) | 8.31 (0–349) |  |
|  |  |  |  |  |  |  |  |  |  |  |
|  |  |  |  |  |  |  |  |  |  |  |
