# Supplementary material for: Low levels of sibship encourage use of larvae in western Atlantic bluefin tuna abundance estimation by close-kin mark-recapture
Source: Sci Rep. 2022 Nov 3;12:18606. doi: 10.1038/s41598-022-20862-9 (PMC9633702; doi:10.1038/s41598-022-20862-9)
Supplement: Supplementary file 2 — Supplementary Information 2. [file 41598_2022_20862_MOESM2_ESM.pdf]

Supplement

A: CKMR as an Unbiased Estimating Equation

There are three steps to the argument:

1. CKMR following Bravington, Skaug, and Anderson (2016) (shown as [14], as per main text) is based around a "pseudo-log-likelihood" that sums the log-likelihoods from pairwise comparisons. The "pseudo" is there because the pairwise comparisons may not be independent: certainly not, if sibship is elevated in the samples.

2. Log-likelihoods lead to Unbiased Estimating Equations, and therefore so does a sum of log-likelihoods, even if (as here) the sum itself is not a true log-likelihood.

3. Unbiased Estimating Equations lead to estimates that are unbiased, at least asymptotically with large enough sample sizes (which is all that can normally be achieved in statistics; the colloquial shorthand is just to say "unbiased", e.g. for Maximum Likelihood Estimates).

The proofs of (2) and (3) are well-known in theoretical statistics, and [14] establishes (1) for CKMR. However, for completeness, we re-derive (2) here for the special case of pairwise CKMR POP comparisons.

Suppose we wish to estimate parameters θ from POP-based CKMR by comparing a sample $\mathcal{A}$ of adults to a sample $\mathcal{J}$ of juveniles; the true, unknown, value of θ is θ_0. Let $p_{aj}(\theta)$ be the demographic probability formula that individual samples a and j are POP (as determined by ERRO principles in [14]), and letY_{aj} be the random variable holding the outcome of their genetic comparison: 1 if POP or 0 if not. Thus by definition $\mathbb{E}[Y_{aj}] = p_{aj}(\theta_0)$. Then the log-likelihood $\Lambda_{aj}(\theta)$ for their comparison is

$$\Lambda_{aj}(\theta; Y) = \log\left(p_{aj}^{Y_{aj}}(\theta)\left(1 - p_{aj}(\theta)\right)^{1-Y_{aj}}\right) \tag{1}$$

and the corresponding score-function for that comparison is (omitting explicit dependence on θ where possible, and writing ∇x for $dx/d\theta$)

$$\nabla\Lambda_{aj}(\theta; Y) = Y_{aj} p_{aj}^{-1} \nabla p_{aj} - (1 - Y_{aj})(1 - p_{aj})^{-1} \nabla p_{aj} \tag{2}$$

The estimate of θ is the root of the pseudo-score estimating equation, i.e. the value $\hat{\theta}_Y$which satisfies

$$\sum \nabla\Lambda_{aj}\left(\hat{\theta}_Y; Y\right) = 0 \tag{3}$$

where summation is over all sampled a's and j's. At the true value θ_0, writing $p_{aj0} \equiv p_{aj}(\theta_0)$ and $\nabla p_{aj0} \equiv \nabla p_{aj}(\theta_0)$, we have

$$\begin{aligned} \mathbb{E}_Y\left[\nabla\Lambda_{aj}(\theta_0; Y)\right] &= \sum\left(\mathbb{E}[Y_{aj}]\, p_{aj0}^{-1} - \frac{1 - \mathbb{E}[Y_{aj}]}{1 - p_{aj0}}\right)\nabla p_{aj0} \\ &= \sum\left(\frac{p_{aj0}}{p_{aj0}} - \frac{1 - p_{aj0}}{1 - p_{aj0}}\right)\nabla p_{aj0} = \sum 0 = 0 \end{aligned} \tag{4}$$

This property holds exactly, regardless of whether the Y_{aj}'s are independent— which they are not, in high-sib CKMR sampling. Thus the CKMR pseudo-score yields an *unbiased* estimating equation in the sense of Godambe and Thompson (1978); this is the basic statistical requirement which implies *asymptotic* unbiasedness (consistency) of $\hat{\theta}$ estimates. For any finite sample size, it is inevitable that most parametrizations of θ will still exhibit bias; for example, cartoon CKMR is unbiased for $1/N$ but not for N itself. However, that bias will diminish at rate m^{-1} in sample size m, whereas the standard error in $\hat{\theta}$ will decline only at rate $m^{-1/2}$. Thus, by the time the sample size is big enough to yield usefully small uncertainty in $\hat{\theta}$, the bias in $\hat{\theta}$ (under some specific parametrization) will generally be negligible, so that it is reasonable to simply say "estimates are unbiased". This is directly relevant to large-population sparse-sampling CKMR, noting that the appropriate definition of "sample size" is typically related to the number of comparisons, i.e. quadratic in the actual number of samples ([14], section 5).

B: Effect of sibship on variance in a simple setting

For this calculation, we consider a large single-sex population of N adults all with same fecundity and same capture probability, where random samples are drawn from juveniles and the adults soon after birth of the former, with adult and juvenile sample sizes m_A and m_J much smaller than N. This is like Figure 1 of [14], but with only one adult sex.

Let $Y_{aj} \in \{0,1\}$ be result of a POP comparison between adult a and juvenile j, with $Y = 1$ if they are a POP and 0 if not. Then $\mathbb{P}[Y_{aj} = 1] = \mathbb{E}[Y_{aj}] = 1/N$; the Poisson likelihood for the comparison is $\log\left(\exp(-1/N) \times N^{-Y_{aj}}/Y_{aj}!\right) = -N^{-1} - Y_{aj}\log N + c$ where c does not depend on N; and the score function (derivative of the log-likelihood) for the comparison is $N^{-2} - Y_{aj}N^{-1}$. The total score for all comparisons between m_J sampled juveniles and m_A adults (technically a "pseudo-score" since the comparisons are not independent under high-sibship sampling) is $m_J m_A N^{-2} - \left(\sum_{a,j} Y_{aj}\right) N^{-1}$), so the reciprocal point estimate from solving the estimating equation "pseudo-score = 0" is given by $1/\hat{N} = \sum Y_{aj}/m_J m_A$. It is easiest to work on the reciprocal-abundance scale, which is linear in the random variables Y, and then to apply asymptotics at the end to get results for $\hat{N}$ itself.

To see the effect of sibship, consider the juvenile samples as fixed, and treat the adult sample as random (note that CKMR gives the same results from either an adult-centric or a juvenile-centric perspective; see Skaug, 2017). For any adult a, whether sampled or not, let W_a be the number of their offspring amongst the m_J juveniles. Then the point estimate can be written as

$$1/\hat{N} = \frac{1}{m_J m_A} \sum_{a \in \mathcal{A}} W_a \tag{5}$$

where $\mathcal{A}$ is the sample of adults. Its variance, arising from the random process of selecting adults independently, is

$$\mathbb{V}\left[1/\hat{N}\right] = \frac{m_A}{(m_J m_A)^2} \mathbb{V}[W] \tag{6}$$

where $\mathbb{V}[W]$ is the variance, across adults in the *population*, of the number of their *sampled* offspring (subscript a dropped for brevity, since all adults have equal sampling probability in this simple model). For

most adults, $W = 0$; for a few, $W = 1$; and because of sib-rich larval sampling, a small proportion have $W > 1$. There is information on $\mathbb{V}[W]$ from the DPG sizes, but those are censored that so that we do not observe cases of $W = 0$, which are the great majority. Let $p_0 = \mathbb{P}[W = 0]$, so that $1 - p_0 = O(1/N) \ll 1$, and define

$$
\begin{aligned}
e_{1+} &\triangleq \mathbb{E}[W|W > 0] \\
e_{2+} &\triangleq \mathbb{E}\left[W^2|W > 0\right] \\
e_1 &\triangleq \mathbb{E}[W] \\
e_2 &\triangleq \mathbb{E}\left[W^2\right]
\end{aligned}
$$

Note that $e_{1+} \geqslant 1$ by definition, and e_{2+} will be somewhat larger but not immense provided that the DPGs are small. If there is no sibship in the juvenile sample, then $e_{1+} = e_{2+} = 1$.

Since there are m_J juveniles drawn from N exchangeable adults, $e_1 = \mathbb{E}[W] = m_J/N$. By definition $e_1 = p_0 \times 0 + (1 - p_0) \times e_{1+}$ so that $1 - p_0 = m_J/(Ne_{1+})$; and similarly $e_2 = p_0 \times 0^2 + (1 - p_0) \times e_{2+}$. On substituting for $1 - p_0$, we have $e_2 = m_J e_{2+}/(Ne_{1+})$. The variance of W is just $\mathbb{V}[W] = e_2 - e_1^2$, but the term $e_1^2 = (m_J/N)^2$ is negligible compared to $e_2 = (m_J/N) \times (e_{2+}/e_{1+})$. Thus

$$
\mathbb{V}[W] \approx e_2 = \frac{m_J}{N} \times \frac{e_{2+}}{e_{1+}} \tag{7}
$$

In the absence of sibship, this reduces to $\mathbb{V}[W] = m_J/N$ and, from equation (2), $\mathbb{V}\left[1/\hat{N}\right] = 1/(m_J m_A N)$.

The quadratic term e_{2+} in equation (7) means that it is not just the *number* of distinct parents which matters, but also their pattern; have a few large DPGs and many small ones is worse for CKMR variance than having more evenly-sized DPGs, even if the total number of distinct parents is the same.

Assuming that male and female adults are equally abundant and are similar in terms of sib-based overdispersion, empirical estimates of e_{2+} and e_{1+} can be computed directly from tabulated non-sex-specific DPG-sizes, as in Table 3 of the main text. For example, in 2017 the actual larval sample size was 317, and we have

$$
\begin{aligned}
\text{Number of DPGs} &= 398 + 89 + 12 + 3 + 2 = 504 \\
\hat{e}_{1+} &= \text{mean DPG size} \\
&= (398 \times 1 + 89 \times 2 + 12 \times 3 + 3 \times 4 + 2 \times 5) / 504 = 634/504 \approx 1.26 \\
\hat{e}_{2+} &= \left(398 \times 1^2 + 89 \times 2^2 + 12 \times 3^2 + 3 \times 4^2 + 2 \times 5^2\right) / 504 = 960/504 \approx 1.91
\end{aligned}
$$

The Variance-Inflation-Factor e_{2+}/e_{1+}, relative to a hypothetical independent sample of 317 juveniles, is $1.91/1.26 \approx 1.51$. Equivalently, the "effective sample size" $m_{J\text{eff}}$ is $317/1.51 \approx 209$. The latter is chosen so that $\mathbb{V}\left[1/\hat{N}\right] = (m_J/m_{J\text{eff}}) \times \mathbb{V}_{\text{naive}}\left[1/\hat{N}\right]$ where the latter is calculated as if the juvenile samples were independent, via $\mathbb{V}_{\text{naive}}\left[1/\hat{N}\right] = 1/(m_J m_A N)$. By asymptotic arguments ("the Delta method"), the same Variance-Inflation-Factor applies to $\hat{N}$ itself, since $\mathbb{V}\left[\hat{N}\right] \approx N^4 \mathbb{V}\left[1/\hat{N}\right]$.

Combining across cohorts

These variance formulae for "cartoon" CKMR apply to one estimate of N based on a single cohort of juveniles. To roughly mimick the multi-cohort aspects of our BFT setup, consider a setting with $C > 1$ juvenile cohorts where the same adult sample is re-used for each juvenile cohort, with N assumed constant. An optimal (linear) combined estimate $1/\hat{N}_{\text{combo}}$ could be made by inverse-variance-weighting the single-cohort estimates $1/\hat{N}_1 \cdots 1/\hat{N}_C$, since samples (and comparisons with adults) from *different* juvenile cohorts are independent. (Again, it makes no difference asymptotically whether we estimate $1/N$ or N, but the former leads to much simpler formulae.) Since variance is inverse to effective sample size by the latter's definition, the inverse-variance-weighted combination will have the same variance as a single sample of size equal to the totals of the effective sample sizes. The proof is pretty obvious, but is included here for completeness:

Let $H_i : i \in \{1 \cdots C\}$ be a set of independent unbiased estimators of some quantity h, with variances kv_i. The inverse-weighted-variance combination of H's is

$$\bar{H} = \frac{\sum H_i/v_i}{\sum 1/v_i}$$

with variance given by

$$\begin{aligned}\mathbb{V}\left[\bar{H}\right] &= \left(\sum 1/v_i\right)^{-2} \sum \mathbb{V}\left[H_i\right]/v_i^2 \\ &= \left(\sum 1/v_i\right)^{-2} \sum k/v_i \\ &= k/\left(\sum 1/v_i\right) \end{aligned} \tag{8}$$

In the "multi-cohort cartoon" case, set $H_i = 1/\hat{N}_i$ and $k = 1/(Nm_A)$ and $v_i = 1/m_{J\text{eff}i}$ so that $\mathbb{V}[H_i] = kv_i = (Nm_A m_{J\text{eff}i})^{-1}$; then $\mathbb{V}\left[1/\bar{N}\right] = (Nm_A \sum_{i=1}^{c} m_{J\text{eff}i})^{-1}$, just as if we had a single sample of effective size $\sum m_{J\text{eff}i}$.

XHSPs

The same approach can be used to study effective sample size in a hypothetical cross-cohort-half-sib-only model. Again, tractability demands a simplified model, and the real point is see how the variance of number of kin-pairs is inflated. Thus we consider just a two-cohort model with zero adult mortality between them, with samples of size m_J from both cohorts collected in the same way so that sibship rates are similar, and with only one parental sex. Also for simplicity, we consider a slight modification of the sampling setup in which the first juvenile sample comprises a Poisson-distributed random number P of DPGs, the ith DPG having size W_{+i1} (the same W as before, restricted to $W > 0$, again with $\mathbb{E}[W_{+i1}] = e_{1+} \,\forall i$). In this setup, the true sample size is actually a random variable with expected value m_J, rather than being completely fixed. Since both the actual sample size and the number of DPGs are large (e.g. hundreds) the distinction is unimportant.

Let Q_i be the number of XHSPs that arise from the ith DPG in the first sample: thus $Q_i = W_{+i1} \times W_{i2}$ with obvious notation, and $W_{+1i} \perp W_{i2}$ (i.e. independence). Write $Q^* \triangleq \sum_i Q_i$ for the total number of XHSPs.

The Law of Total Variance gives:

$$\mathbb{V}[Q^*] = \mathbb{E}_P[\mathbb{V}[Q^*|P]] + \mathbb{V}_P[\mathbb{E}[Q^*|P]] \tag{9}$$

By definition, since the expected sample size in cohort 1 is m_J and the number of DPGs is Poisson, we have:

$$m_J = \mathbb{E}[P] \times \mathbb{E}[W_+] = \mathbb{E}[P] \times e_{1+}$$
$$\implies \mathbb{E}[P] = \mathbb{V}[P] = m_J/e_{1+}$$

Writing $v \triangleq \mathbb{V}[W]$ and $v_+ = \mathbb{V}[W_+]$, and using results from the POP calculations, we have

$$\begin{aligned}
\mathbb{V}[Q^*|P] &= \mathbb{V}\left[\sum_{i=1}^{P} W_{+i1} W_{i2}\right] \\
&= P\mathbb{V}[W_{+1i} W_{i2}] \\
&= P\left(e_{2+}e_2 - e_{1+}^2 e_1^2\right) \\
&= P\left(e_{2+}\frac{m_J}{N} \times \frac{e_{2+}}{e_{1+}} - e_{1+}^2 \frac{m_J^2}{N^2}\right) \\
&\approx P\left(\frac{e_{2+}^2 m_J}{N}\right) \\
\implies \mathbb{E}_P[\mathbb{V}[Q^*|P]] &= \frac{m_J^2 e_{2+}^2}{N e_{1+}}
\end{aligned} \tag{10}$$

with the approximation, as in the POP case, being that $e_1^2 \ll e_2$ since both e_1 and e_2 are $O\left(N^{-1}\right)$. Also

$$\begin{aligned}
\mathbb{E}[Q^*|P] &= P \times \mathbb{E}[Q_i] \\
&= P \times \mathbb{E}[W_{+1i}] \times \mathbb{E}[W_{2i}] \text{ since } W_{+1i} \perp W_{2_i} \\
&= P e_{1+} e_1 = P\frac{e_{1+} m_J}{N} \\
\implies \mathbb{V}_P[\mathbb{E}[Q^*|P]] &= \frac{m_J}{e_{1+}}\left(\frac{e_{1+} m_J}{N}\right)^2 = \frac{m_J^3 e_{1+}}{N^2}
\end{aligned}$$

Using equation (9), this gives

$$\begin{aligned}
\mathbb{V}[Q^*] &= \frac{m_J^2}{N}\left(\frac{e_{2+}^2}{e_{1+}} + \frac{m_J}{N} e_{1+}\right) \\
&\approx \frac{m_J^2}{N} \times \frac{e_{2+}^2}{e_{1+}}
\end{aligned} \tag{11}$$

since $m_J \ll N$ for a large population with sparse sampling *sensu* [14]. For the no-within-cohort sib case where $e_{1+} = e_{2+} = 1$, this gives m_J^2/N as we would expect. The XHSP Variance-Inflation-Factor is e_{2+}^2/e_{1+} which exceeds the POP VIF in equation (7) by a factor e_{2+}.

For WBFT as summarized in Table 3 of the main text, the effective sample size for an XHSP-only model would become 129.8 per year if all cohorts were sampled like 2016, or 109.9 per year if all were 2017-like. Unlike the POP-only case, 2017-style targeted sampling would be slightly *less* powerful than grid sampling if an XHSP-

only model was contemplated. However, we are not proposing an XHSP-only model for W BFT, since it would only make sense to use both POPs and XHSPs together.

C: Distinct Parent Group algorithm

This algorithm partitions a sample of juveniles into Distinct Parent Groups (DPGs), each group containing all sampled progeny from one unknown adult. The inputs are a list of HSPs, a list of FSPs, and a list of "unrelated" samples that are not in HSPs nor FSPs. This algorithm alone cannot reveal the sex of each DPG's parent; however, once the DPGs are identified, parental sex can be gleaned by comparing mtDNA within DPGs (since members of a maternally-linked DPG must all have the same mtDNA haplotype, whereas in a paternally-linked DPG there will be multiple haplotypes with high probability).

The algorithm assumes that each adult is always the same sex (no hermaphrodites), and that the pairwise kinships are complete and internally consistent. The kinship assumptions and completeness can be checked within the algorithm, at least up to a point; it would clearly be impossible, for example, to detect one single missing sib of an otherwise-unrelated animal. Those checks are omitted here for brevity.

The key to the algorithm is that the *half*-sibs of any animal must be partitioned into exactly two subsets, one sharing the same Mother and one all sharing the same Father, with one subset possibly empty. All the members of that Mother-linked subset must also share a Mother with each other, and ditto for the Father-linked set. Full-sibs complicate the picture, so are removed at the start and then re-inserted later.

1. Start with an empty list of DPGs.
2. Find all groups of Full-sibs (so that all members of each such group are FS of each other) and pick one Representative from each, arbitrarily.
3. In the HSPs, replace all occurrences of other members of each Full-sib-group by that group's Representative.
4. Reduce the modified HSPs to unique pairs. Now there cannot be any Full-sibs left in the HSPs; each animal therein shares at most one parent with any other.
5. For each not-yet-checked animal i in the HSPs:
   (a) Find its first HSP, j_i, and colour j_i Red
   (b) For k_i being the 2nd, 3rd, etc HSs of i:
      i. check if j_i and k_i are HSP. If so, colour k_i Red; if not, colour k_i Blue.
   (c) All HS of i are now either Red or Blue (there may be no Blue ones). Red ones share one of i's parents, and Blue ones share the other. Add i to both the Red and Blue groups.
   (d) Add the Red and the Blue groups to the list of DPGs (discarding colour).
   (e) Mark j_i and all k_i as "checked", so they do not need to be examined in subsequent iterations (although they can be scrutinized for consistency).
6. Wherever a Full-sib Representative occurs in a DPG, replace it by its entire Full-sib group.
7. For each Full-sib group whose Representative is *not* in any of the HSPs:

(a) duplicate that Full-sib group, and add both copies to the list of DPGs (one copy shares a Mother, and the other shares a Father).

8. Each Unrelated animal (i.e. not part of any FSP nor any HSP) forms two gDPGs of size 1; it shares a Mother with no-one, and a Father with no-one. Add all these groups to the DPG list.

References

Bravington, Mark V., Hans J. Skaug, and Eric C. Anderson (May 2016). "Close-Kin Mark-Recapture". In: *Statistical Science* 31.2, pp. 259–274. DOI: 10.1214/16-STS552. URL: https://doi.org/10.1214/16-STS552.

Godambe, V.P. and M.E. Thompson (1978). "Some aspects of the theory of estimating equations". In: *Journal of Statistical Planning and Inference* 2.1, pp. 95–104. ISSN: 0378-3758. DOI: https://doi.org/10.1016/0378-3758(78)90026-5.

Skaug, Hans J. (2017). "The parent-offspring probability when sampling age-structured populations". In: *Theoretical Population Biology* 118, pp. 20–26. ISSN: 0040-5809. DOI: https://doi.org/10.1016/j.tpb.2017.09.001. URL: http://www.sciencedirect.com/science/article/pii/S0040580917300138.
